# Nasal Delivery to the Brain: Harnessing Nanoparticles for Effective Drug Transport

**DOI:** 10.3390/pharmaceutics16040481

**Published:** 2024-04-01

**Authors:** Shivani Gandhi, Divyesh H. Shastri, Jigar Shah, Anroop B. Nair, Shery Jacob

**Affiliations:** 1 Department of Pharmaceutics, K. B. Institute of Pharmaceutical Education and Research, A Constituent College of Kadi Sarva Vishwavidyalaya, Sarva Vidyalaya Kelavani Mandal, Gh-6, Sector-23, Kadi Campus, Gandhinagar 382023, Gujarat, India; shivani.g-pharmacy@msubaroda.ac.in; 2 Department of Pharmaceutics, Institute of Pharmacy, Nirma University, Ahmedabad 382481, Gujarat, India; jigar.shah@nirmauni.ac.in; 3 Department of Pharmaceutical Sciences, College of Clinical Pharmacy, King Faisal University, Al-Ahsa 31982, Saudi Arabia; anair@kfu.edu.sa; 4 Department of Pharmaceutical Sciences, College of Pharmacy, Gulf Medical University, Ajman 4184, United Arab Emirates; dr.sheryjacob@gmu.ac.ae

**Keywords:** nose to brain, nanocarriers, nanogels, formulation strategies, blood–brain barrier

## Abstract

The nose-to-brain drug-delivery system has emerged as a promising strategy to overcome the challenges associated with conventional drug administration for central nervous system disorders. This emerging field is driven by the anatomical advantages of the nasal route, enabling the direct transport of drugs from the nasal cavity to the brain, thereby circumventing the blood–brain barrier. This review highlights the significance of the anatomical features of the nasal cavity, emphasizing its high permeability and rich blood supply that facilitate rapid drug absorption and onset of action, rendering it a promising domain for neurological therapeutics. Exploring recent developments and innovations in different nanocarriers such as liposomes, polymeric nanoparticles, solid lipid nanoparticles, dendrimers, micelles, nanoemulsions, nanosuspensions, carbon nanotubes, mesoporous silica nanoparticles, and nanogels unveils their diverse functions in improving drug-delivery efficiency and targeting specificity within this system. To minimize the potential risk of nanoparticle-induced toxicity in the nasal mucosa, this article also delves into the latest advancements in the formulation strategies commonly involving surface modifications, incorporating cutting-edge materials, the adjustment of particle properties, and the development of novel formulations to improve drug stability, release kinetics, and targeting specificity. These approaches aim to enhance drug absorption while minimizing adverse effects. These strategies hold the potential to catalyze the advancement of safer and more efficient nose-to-brain drug-delivery systems, consequently revolutionizing treatments for neurological disorders. This review provides a valuable resource for researchers, clinicians, and pharmaceutical-industry professionals seeking to advance the development of effective and safe therapies for central nervous system disorders.

## 1. Introduction

The nose-to-brain medication-delivery system is a revolutionary concept in pharmaceutical research, offering a new pathway to transport therapeutic substances directly from the nasal cavity to the brain [1]. This novel strategy takes advantage of the complex and particular structure of the nasal canal, utilizing its unique link to the brain via the olfactory and trigeminal nerve pathways [2]. This delivery technology overcomes the challenging blood–brain barrier (BBB), enabling the direct delivery of drugs to specific areas of the brain, a major hurdle in standard drug administration. This method has great promise for treating a range of neurological illnesses, such as Alzheimer’s, Parkinson’s, and brain cancers, among others [3]. The non-invasive characteristic and ability to distribute drugs with precision and localization represent a significant advancement in the field of neuropharmacology. Beyond enhancing drug delivery, this technique has the potential to optimize therapeutic outcomes, reduce systemic adverse effects, and address unmet medical needs in the field of neurological therapies [4]. The convergence of anatomical knowledge, pharmaceutical advancements, and precise treatment methods underscores the considerable potential of the nose-to-brain delivery system in transforming the field of neurological treatments [5].

## 2. Nasal Anatomy and Physiology

The nasal architecture is a complex system that includes both the external nose and the nasal cavity, both of which are crucial for respiratory function. The external nose, consisting of cartilage and bone, functions as the visible opening for inhaled air. Upon entering the nasal cavity, which is partitioned by the nasal septum, it serves as the primary pathway for respiratory function [6,7,8]. The respiratory epithelium, which lines the nose passages, has specialized cilia that function as tiny brooms, effectively removing particulate debris and foreign particles from inhaled air. Concurrently, blood vessels in the mucosal membrane aid in air conditioning by heating and moisturizing the inhaled air.

The olfactory epithelium, located deep inside the nasal cavity, is responsible for the sensation of smell. This area has a neural network that enables the identification and understanding of different smells. The olfactory system is essential in our sensory perceptions and has the ability to impact our entire state of well-being [9,10,11,12]. Moreover, the nasal architecture includes junctional complexes that are responsible for preserving the integrity of the nasal epithelial barrier. These complexes play a vital role in inhibiting the infiltration of detrimental microorganisms and guaranteeing the optimal functioning of the respiratory system [13,14]. To summarize, the complex structure of the outer nose and nasal cavity, along with the respiratory and olfactory epithelia and the presence of junctional complexes, all work together to effectively condition, filter, and process the air that humans breathe in through the nose, as shown in Table 1 [14,15,16,17].

## 3. Considerations in Constructing Nose-to-Brain Drug-Delivery Systems

Constructing nose-to-brain drug-delivery systems requires careful consideration of several key factors to achieve targeted and efficient drug delivery to the central nervous system (CNS) [21]. An in-depth understanding of nasal anatomy and physiology is paramount, as factors such as mucosal permeability, surface area, and blood supply influence the absorption and distribution of drugs administered through the nasal route [22]. Hence, researchers should also account for anatomical differences among species in which formulations are being evaluated, including nasal pH, blood circulation, enzyme activity, mucociliary clearance, disparities in dosing volumes, etc. [23]. Additionally, the physicochemical properties of the drug, including size, lipophilicity, and molecular weight, etc. must align with the unique characteristics of the nasal mucosa to facilitate effective penetration and transport to the brain. Formulation design is crucial in this process, with considerations for stability, bioavailability, and sustained release to optimize therapeutic outcomes [24]. Various delivery systems, such as solutions, suspensions, gels, nanoparticles, etc., can be employed based on the specific requirements of the drug and the desired therapeutic effects. Overall, successful nose-to-brain drug delivery hinges on a comprehensive integration of these considerations, aiming for an intricate balance that maximizes drug efficacy while minimizing potential side effects [25].

## 4. Mechanism of Nose-to-Brain Delivery

Nose-to-brain drug delivery encompasses many processes that promote the transportation of medicines from the nasal cavity to the brain (Table 2). The nasopharyngeal space-to-brain pathways play a pivotal role in facilitating the transportation of drugs from the nasal cavity to the brain [26].

**Figure 1:**
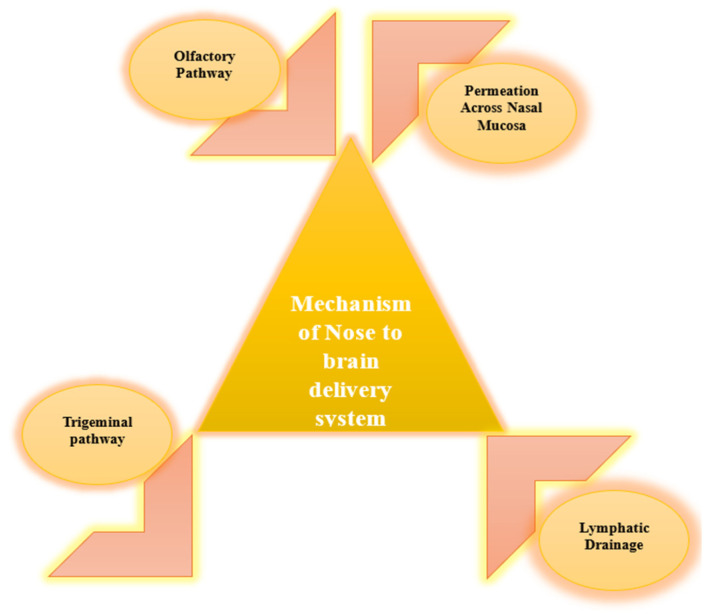
Mechanism of nose-to-brain delivery.

Four routes involved in nose-to-brain delivery (Figure 1) are briefed below. Olfactory pathway: One of the main routes includes the olfactory pathway, where medicines may circumvent the BBB by directly entering the brain via the olfactory epithelium [40]. The drugs are conveyed via the olfactory nerves (along axons and via nerve bundles that cross the cribriform plate), ultimately reaching the olfactory bulb and further progressing to deeper parts of the brain [41].

Trigeminal pathway: When drugs are given via the nose, they may interact with the nerve ends of the trigeminal nerve found in the nasal mucosa [42]. These neurons provide a pathway for drugs to reach other sections of the brain, such as the brainstem and other areas of the CNS, without going through the BBB.

Permeation across nasal mucosa: Substances may also permeate the nasal mucosa, gaining access to the body’s circulatory system and ultimately reaching the brain via the bloodstream [43]. Nevertheless, this pathway may require traversing the BBB, which may restrict the penetration of some substances into the brain.

Lymphatic drainage: Several studies indicate that nasal medication administration may use lymphatic drainage channels, possibly enabling pharmaceuticals to circumvent the BBB and access the brain interstitium via the glymphatic system [44].

## 5. Advantages, Challenges, and Barriers in Nose-to-Brain Drug Delivery

### 5.1. Advantages

#### 5.1.1. Targeted Administration

Nose-to-brain medication delivery bypasses the BBB by targeting the brain via the nasal cavity. Targeted administration uses nanoparticles or liposomes to deliver drugs to the brain. Nanoparticles or liposomes may be modified with ligands or surface changes to bind to nasal mucosa receptors or transporters [34]. This targeting improves drug-loaded nanoparticle absorption and transport across the nasal epithelium to desired brain areas. Researchers can coat nanoparticles with ligands that attach to nasal epithelial receptors. Nanoparticles may enter cells and traverse the nasal epithelium into the brain via receptor-mediated endocytosis [35].

#### 5.1.2. Enhanced Bioavailability

To increase the effectiveness of medication delivery from the nose-to-brain, it is necessary to strategically use nanocarriers to overcome biological obstacles and boost the transportation of drugs to the CNS. Nanocarriers, including nanoparticles and liposomes, have several benefits in terms of medication encapsulation, stability, and precise delivery to specific targets. Their diminutive size enables effective transportation over the nasal mucosa, hence enhancing surface area contact and optimizing medication absorption [36].

Nanocarriers provide a means of safeguarding pharmaceuticals from enzymatic degradation, reducing drug breakdown or elimination, preserving drug stability and improving their solubility, therefore tackling the difficulties arising from the physicochemical characteristics of certain pharmaceutical actives. Moreover, these carriers may be specifically designed to have mucoadhesive characteristics, which enhance their ability to stay on the nasal mucosa for an extended period and facilitate the continuous release of medication with improved therapeutic response [36].

The systemic route serves as an indirect means for nose-to-brain delivery, entailing the direct absorption of the nanocarriers into the blood stream from the nasal respiratory epithelium or through the trigeminal nerve pathway or lamina propria adsorption from perivascular and lymphatic spaces [2,22]. In the case of vascular pathways, firstly the nanocarriers have to enter the nasal cavity and withstand elimination processes mediated by enzymes and mucociliary clearance. Subsequently, the carriers reach the systemic circulation and reach the brain parenchyma by crossing the BBB [45]. However, entry into the systemic circulation does not automatically entail the carrier being directed towards the BBB, even if it possesses the capability to traverse it [46]. Typically, the cellular uptake of nanoparticles is believed to depend on factors like concentration, particle size, surface potential, surface functionalization, and additional surface features like lipophilicity [47]. The performance of nanocarriers at the biological interface can be differentiated based on electrostatic interactions, mucoadhesion, permeation enhancement, and brain targeting via specific absorption mechanisms [48]. These factors guide the drug through intricate and limited transport pathways from the nasal cavity to systemic circulation or even the CNS, influencing nose-to-brain transport. Hence, the selection of ideal nanocarriers is of utmost importance to overcome the formulation as well as drug-delivery challenges for systemic nasal or nose-to-brain drug delivery. In general, one should consider the physicochemical properties, stability, mechanism of action, pharmacodynamics, and potency of the drug while selecting the nanocarrier. It has been described that nanoparticle size plays a crucial role in their absorption into the systemic circulation following intranasal application. However, nanoparticles with sizes less than 200 nm are considered suitable for nose-to-brain delivery. Furthermore, the degree of drug transportation from the nasal cavity to systemic circulation can vary considerably, ranging from nearly complete absorption to less than 1% of the intended dose, which depends mainly on the availability of efficient formulations and carriers [9].

Surface changes of nanocarriers enhance their efficacy in delivering drugs from the nose to the brain. PEGylation, as an example, may augment biocompatibility and diminish clearance by the reticuloendothelial system, thereby extending circulation duration and enhancing medication bioavailability. The attachment of ligands to the nanocarriers allows for active targeting by selectively identifying receptors on nasal epithelial cells, resulting in the improved absorption and transportation of drugs across the BBB. In addition, the use of nanocarriers enables the precise regulation of release patterns, guaranteeing a prolonged therapeutic impact and reducing variations in drug levels. Nanocarriers possess a high degree of adaptability in their design and may be customized to meet specific requirements. This characteristic makes them a very promising approach for strengthening the transport of drugs from the nose to the brain. By doing so, they can improve the availability of drugs in the body and enhance the effectiveness of treatments for neurological disorders [36]. These nanoparticles effectively release the therapeutic payload in the brain, increasing drug concentration in targeted brain locations while limiting systemic exposure. Nanoparticles may cross the BBB and reach greater medication concentrations in brain tissue, thus increasing bioavailability [49].

The improved delivery mechanism potentially enhances the absorption of drugs by the brain and may lower the dose, reducing systemic adverse effects. This targeted approach shows how nanoparticle-based formulations for nose-to-brain drug-delivery systems can revolutionize neurological disorder treatments by improving bioavailability and minimizing systemic impact [36].

#### 5.1.3. Reduced Systemic Side Effects

Visualize the use of an improved nose-to-brain delivery apparatus for the administration of a groundbreaking drug directly to the CNS via the nasal route. Due to their ability to circulate throughout the body, systemic medicines often result in significant side effects. However, this novel technique specifically targets the brain, avoiding systemic circulation and reducing medication exposure to other tissues and organs. The tailored delivery technique, implemented in nose-to-brain drug-delivery systems with the use of nanocarriers, presents a sophisticated strategy to significantly minimize systemic medication exposure and mitigate peripheral tissue and organ side effects. By harnessing the unique properties of nanocarriers, such as nanoparticles and liposomes, this approach enables targeted drug delivery directly to the nasal mucosa. By doing so, it circumvents the systemic circulation and the BBB, thereby reducing the overall systemic exposure of the medication. This precise and localized delivery to the CNS allows for therapeutic efficacy at lower doses, minimizing the risk of adverse effects on peripheral tissues and organs. The small size of nanocarriers enhances their ability to traverse the nasal mucosa efficiently, while controlled release mechanisms contribute to sustained drug levels in the brain. Consequently, this tailored delivery technique not only optimizes drug efficacy for neuropharmaceutical interventions but also addresses a critical concern by substantially lowering the potential for unwanted side effects in peripheral tissues. Thus, patients may encounter less systemic adverse effects including gastrointestinal problems, liver damage, and cardiovascular issues. This technique reduces systemic exposure and off-target effects, improving medication safety and acceptability. Nose-to-brain delivery systems maximize drug efficacy and minimize systemic side effects while improving neurological-disorder treatment safety [50].

#### 5.1.4. Rapid Onset of Action

The pursuit of a rapid onset of action in nose-to-brain drug-delivery systems is paramount for addressing acute neurological conditions. By leveraging the direct nasal route to the CNS, nanocarriers such as nanoparticles and liposomes play a pivotal role in achieving swift therapeutic effects [50]. The inherently rapid absorption through the nasal mucosa, combined with the avoidance of systemic circulation and the BBB, enables a more immediate delivery of drugs to the brain. This expedited transport facilitates a quicker onset of pharmacological activity, crucial for treating conditions where prompt intervention is essential. The small size and specialized formulations of these nanocarriers enhance their ability to efficiently navigate the nasal passages, ensuring a rapid and targeted delivery of drugs for improved patient outcomes in acute neurological scenarios [51].

#### 5.1.5. Non-Invasive Route

A targeted nose-to-brain delivery system is ideal for patients who are in the early-stage of Parkinson’s disease and need non-invasive, patient-friendly therapy. This novel therapy uses the non-invasive nasal route for the delivery of the drug [52]. The nanoparticle-encapsulated medication effectively crosses the nasal mucosa and enters the brain. This non-invasive approach is convenient and gives patients a more pleasant and accessible administration route. By eliminating injections and operations, this strategy improves patient compliance and acceptability, especially in chronic illnesses where long-term treatment adherence is vital. The nasal-administration system’s non-invasiveness reduces patient worry and discomfort, making therapy more pleasant. This non-invasive nose-to-brain delivery system might revolutionize neurological disease therapies by delivering therapeutic compounds directly to the brain in a simple, pleasant, and patient-centric manner [52].

### 5.2. Challenges and Barriers for Nose-to-Brain Drug Delivery and Its Solutions

#### 5.2.1. Clearance of the Mucociliary Tract

The nasal cavity’s mucociliary clearance process presents considerable obstacles to medication-delivery systems targeting the brain via the nose. The nasal epithelium is lined with cilia, constituting a natural defensive mechanism that continuously moves mucus to cleanse the respiratory system of foreign particles and infectious and toxic chemicals. Nevertheless, this effective clearance mechanism also influences the retention and duration of pharmacological compounds in the nasal cavity, hence affecting their absorption and transportation to the brain [53].

Drugs administered via the nose are quickly cleared by the movement of mucus, which lowers the amount of time they stay on the nasal lining. This limits the opportunity for the drug to be absorbed into the brain. Moreover, this method may rapidly eliminate big particles or formulations that are not specifically designed to adhere to mucosal surfaces, thus impeding their ability to effectively reach the brain [54].

This problem requires the development of formulations or carriers that can overcome the process of mucociliary clearance, maybe by improving the ability to stick to the nasal mucosa or by modifying the characteristics of the medication to avoid quick removal. Researchers are now investigating strategies such as using mucoadhesive polymers, nanosized carriers, or other probable alterations that increase the duration of drug retention in the nasal cavity. These approaches aim to overcome the hurdles associated with drug clearance and enhance the effectiveness of drug-delivery systems targeting the brain via the nose. Developing strategies to circumvent or minimize the effects of mucociliary clearance is a vital component in improving the efficiency and efficacy of drugs intended for delivery to the brain via the nasal pathway [53,54]. Mucopenetrating drug carriers are also one of the strategies amongst them designed by macromolecular building blocks based on PEG or thiolated polymers, by using zeta potential-shifting ability or high charge density and designing formulations based on mucolytic properties [55].

#### 5.2.2. Drug Attributes

The attributes of pharmaceuticals provide substantial obstacles to the efficacy of nose-to-brain drug-delivery systems. The size of molecules is a critical factor since molecules with large molecular weight typically have difficulties in effectively entering the nasal mucosa, hindering their absorption and subsequent transportation to the brain. Furthermore, the equilibrium between a drug’s affinity for water and its affinity for lipids affects its capacity to cross the nasal epithelium. Drugs with a strong affinity for water may have difficulties in traversing barriers that are rich in lipids, while compounds with a strong affinity for lipids may experience problems due to their low solubility in water, which might impact their absorption process [56,57]. Furthermore, the chemical stability of medicines in the nasal environment poses challenges. Certain drugs may degrade when they come into contact with nasal enzymes or the acidic pH in the nasal cavity, which reduces their efficiency while being transported to the brain. The metabolism occurring in the nasal mucosa might also diminish the quantity of active medication accessible for absorption by the brain [58]. Efflux transporters in the nasal mucosa exacerbate the situation by aggressively expelling drugs, so restricting their absorption and reducing the likelihood of their reaching the brain in enough amounts. Furthermore, the quantity of medication that can be efficiently administered via the nasal route may be influenced by dose restrictions related to the volume of the nasal cavity or limits in the formulation [56,58].

In order to tackle these issues, techniques are designed to improve formulations by taking into account the physicochemical qualities and stability, while also finding solutions to the restrictions imposed by the nasal architecture and physiology. Researchers are investigating advancements in nanotechnology, carrier systems, and chemical alterations to increase the effectiveness of drug-delivery systems that target the brain via the nose. The goal of such an approach is to optimize drug absorption, stability, and transport in order to achieve more effective brain targeting [58].

#### 5.2.3. Nasal Architecture

The diversity in nasal structure across people presents considerable obstacles to the creation of dependable medication delivery systems from the nose to the brain. The complex anatomy of the nasal cavity, which includes changes in the thickness of the mucosal lining, the surface area, and the patterns of airflow, affects the reliability and effectiveness of medication absorption and transportation to the brain [43]. Variations in nasal architecture may have a substantial impact on the distribution and retention of drugs in the nasal cavity. Differences in the thickness and properties of the mucosal lining in various parts of the nasal cavity may impact how quickly drugs are absorbed, which may have an effect on their availability in the body and their ability to reach the brain. Furthermore, variations in the airway flow or the ability to breathe through the nose among people may affect how long the drug stays in the nasal cavity [59].

Furthermore, structural variations such as deviations in the nasal septum, soft growths on the nose lining known as nasal polyps, or other abnormal conditions might also hinder the process of medication distribution and absorption. These anatomical differences might cause unequal distribution of drugs or impede the interaction between drug formulations and the nasal mucosa, which can impact drug absorption and subsequent delivery to the brain. To tackle this difficulty, it is necessary to formulate medication-delivery systems that can accommodate and adjust to the many anatomical differences in the nasal passages of people. It is essential to customize formulations or systems in order to enhance the deposition and absorption of drugs, taking into account these anatomical variations. This is critical for ensuring the reliable and efficient transport of drugs from the nose to the brain in different groups of patients. The nasal-deposition profiles play important role in the clinical performance of nose-to-brain delivery. The deposition pattern is one of the key factors in the making of efficient nasal formulations. The development of optimized delivery protocols with administration information for site-specific delivery to the olfactory region and can reduce off-target drug loss [60]. To improve the reliability and effectiveness of nasal drug-delivery systems, it is crucial to use strategies that provide a consistent distribution of the medication and extended contact with the nasal mucosa, even in the presence of anatomical variations [59,61].

#### 5.2.4. Formulation Stability

The effectiveness of nose-to-brain medication-delivery systems is critically dependent on the stability of the formulation. The nasal environment, which is characterized by fluctuations in pH, enzyme activity, and mucosal turnover rates, poses challenges in preserving the stability of medication formulations designed for brain targeting [62]. Nasal medication formulations are susceptible to enzymatic degradation by enzymes like epoxide hydrolase, aldehyde hydrogenase, carbosylesterase, and glutathione S-transferase, etc., found in the nasal mucosa, which may result in the metabolism of active pharmaceutical components. Moreover, the pH fluctuations in the nasal cavity, which may range from mildly acidic to neutral, might influence the stability of formulations that are susceptible to pH alterations. This has the potential to undermine their effectiveness while they are being transported to the brain. Additional intricacies develop due to the mucosal turnover rate, whereby fast clearance systems in the nasal canal may restrict the duration of formulation absorption. The high rate of turnover may provide a challenge to the formulation’s ability to stick to the nasal mucosa, which can affect the retention and absorption of the medicine.

To overcome these stability issues, it is necessary to design medication formulations that are resistant to enzyme breakdown i.e., particle encapsulations (liposomes or polymeric nanoparticulate systems, etc.), pH variations, fast mucociliary clearance, i.e., mucoadhesive polymeric nanoparticles, etc. The process of encapsulation provides a protective barrier for pharmaceuticals, preventing them from being broken down by enzymes in the nasal mucosa. This results in a longer duration of drug presence and increases the chances of the drugs reaching the brain without being damaged. In addition, nanoparticulate formulations combined with enzyme inhibitors, penetration enhancers, nasal absorption enhancers, mucoadhesive polymers, etc., could increase the drug permeation to the brain.

Formulations should be specifically engineered to endure the constantly changing circumstances of the nasal environment, guaranteeing the long-lasting stability and intactness of drugs throughout their passage through the nasal cavity. The use of protective excipients, encapsulating technologies, or chemical alterations, is intended to improve the stability of the formulation, hence maintaining the drug’s effectiveness and availability for successful transport from the nose to the brain. Nanoparticles of sizes greater than 100 nm are not suitable, as their size exceeds that of the axons present in the filia olfactoria for intraneuronal route absorption [30,63]. In brief, nanotechnology-based formulations are most suitable for protecting the drug, improving the interaction with the olfactory region and attaining the stability of these formulations and the efficiency of medication delivery to the brain via the nasal route [64].

#### 5.2.5. Delivery to Target Sites

The delivery of therapeutic substances to specific locations inside the brain presents significant difficulties in drug-delivery systems that target the nose-to-brain route. The intricate structure of the brain, characterized by many areas and barriers, requires accurate and focused administration of medication to effectively treat neurological disorders [64]. The delivery of drugs to particular regions of the brain via the nasal route is hindered by challenges associated with the BBB and the complex nature of brain structures. The BBB, while essential for safeguarding the brain, presents a significant obstacle by selectively impeding the entry of substances, including drugs, into the brain tissue. Many drugs have significant difficulties in crossing this barrier, which hinders their capacity to effectively reach specific areas of the brain at therapeutic levels [65].

Moreover, the successful administration of drugs for particular neurological diseases in the brain necessitates overcoming obstacles that extend beyond the BBB. The presence of variations in brain architecture, diverse neural routes, and the need to access brain areas that are located deep inside the brain add additional layers of complexity. The key problem lies in ensuring that drugs effectively reach the intended areas in enough quantities to have therapeutic effects. To tackle these issues, it is necessary to formulate delivery methods that can circumvent or overcome the obstacles presented by the BBB and accurately focus on specific parts of the brain. Nanoparticles’ diminutive dimensions allow for enhanced interactions with the nasal epithelium, resulting in improved medication penetration across mucosal barriers. This increased contact enables a more effective absorption of drugs, boosting their transportation to the brain [1]. Techniques such as surface modifications, nanocarriers with particular ligands, or novel formulations are used to improve the ability of drugs to enter the brain and interact with specific targets. These alterations improve the nanoparticles’ attraction to receptors or transporters in the nasal mucosa, making their absorption and subsequent transportation to specific brain regions more efficient [45]. To fully harness the promise of nose-to-brain drug-delivery systems in treating neurological illnesses, it is essential to address and overcome the obstacles associated with delivering medicines accurately to the targeted areas of the brain [65].

## 6. Direct Transport of Drugs in Solution from Nose to Brain

The direct delivery of drugs in solution from the nasal cavity to the brain entails the transportation of drug molecules via the nasal mucosa, allowing them to reach the brain without being absorbed into the bloodstream or crossing the BBB. This pathway avoids the process of systemic circulation, enabling medicines to immediately reach the brain via the nasal cavity [46]. When drugs are introduced as solutions or suspensions via the nasal route, they can easily make contact with the nasal mucosa. The nasal epithelium, consisting of a porous membrane, enables the absorption of these drugs into circulation and also offers a direct pathway for some pharmaceuticals to overcome the BBB [45]. The drug’s capacity to penetrate the nasal epithelium and reach the brain is influenced by factors such as its physicochemical qualities, including molecular weight, lipophilicity, and solubility. Moreover, the existence of transporters or receptors in the nasal mucosa might expedite the direct transportation of certain drugs to the brain [45,46].

The intranasal route of transportation of the drug improves the efficacy of the drug, making it available specifically to the brain, improving site-specificity and avoiding systemic side effects. Nevertheless, some drugs may encounter obstacles while attempting to employ this pathway, either owing to the nasal epithelium or the need to avoid the BBB. The objective of strategies incorporating formulation adjustments or specialized drug designs is to optimize the effectiveness of this direct transport pathway for the efficient delivery of drugs from the nose to the brain [45].

## 7. Nanoparticles for Nose-to-Brain Drug Delivery

**Figure 2:**
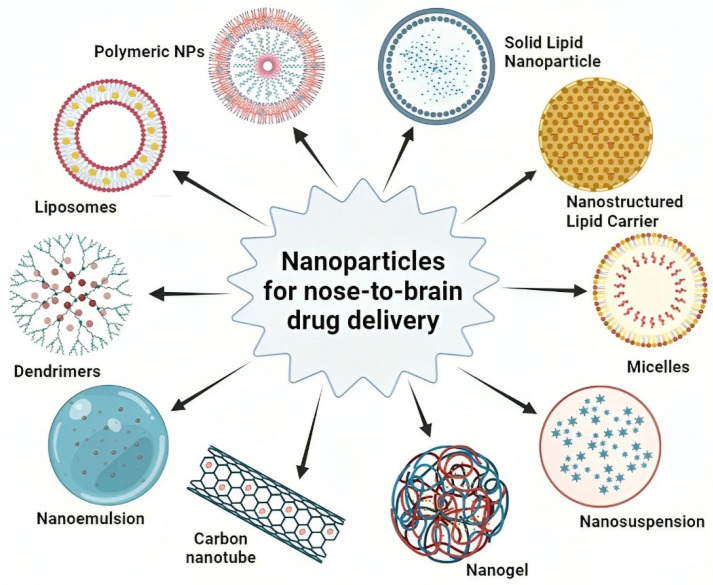
Different nanoparticles used in nose-to-brain drug delivery. Created with BioRender.com.

Nanoparticles are an advanced method for delivering drugs from the nose to the brain. They provide a flexible platform to overcome different obstacles in transferring therapeutic substances to the brain. These minuscule particles, usually measuring between 1 and 100 nanometers in size, have great promise for improving the stability of drugs, regulating their release [66], and delivering them specifically to designated parts of the brain [67]. Endocytic routes are frequently used by nanocarriers smaller than 100 nm to promote mucosal and transcellular transit. Nanoparticles provide precise control over the rate at which drugs are released, guaranteeing a consistent and regulated administration of medication to the brain. The controlled release pattern of this carrier ensures that therapeutic concentrations are consistently maintained, enhancing the effectiveness of the treatment and perhaps allowing for a decrease in the frequency of doses. Their ability to interact well with living tissues and their lack of toxicity make them safe, reducing any negative impact on the nasal lining and the brain. A variety of formulations, such as liposomes, polymeric nanoparticles, solid lipid nanoparticles (SLN), and dendrimers, provide flexible platforms for enclosing drugs, enabling customized approaches in drug administration which are shown in Figure 2. In general, nanoparticles show great potential in the field of nose-to-brain medication delivery. They are constantly being improved and developed to overcome obstacles and enhance the effective and precise transport of drugs to the brain for the treatment of neurological illnesses [68]. Various nanoparticles with their characteristics for nose-to-brain drug-delivery systems are described in Table 3.

### 7.1. Liposomes for Nose-to-Brain Delivery

Liposomal formulations have gained tremendous popularity as they are biocompatible, non-toxic, and deliver both hydrophilic and hydrophobic molecules [31,95]. Liposomes, as adaptable lipid-based nanoparticles (Figure 2), have the potential to enhance medication transport from the nose to the brain. This is attributed to their compatibility with biological systems, capacity to encapsulate diverse medicines, and ability to target particular areas of the brain [31]. Ex vivo studies through sheep nasal membranes for drugs (tacrine and lamotrigine) demonstrated enhanced nasal permeability from liposomal formulations, suggesting their utility in nose-to-brain drug delivery [96,97]. Bender et al. demonstrated that glial-derived neurotrophic factor, when delivered through liposomal formulations, reached its highest levels in the olfactory bulb region of the brain within 1 h of administration [98]. Slower systemic absorption was observed with the H102 liposomal formulation administered intranasally, which remained unabsorbed when administered intravenously due to its inability to cross the BBB [99].

The brain levels of lipophilic and amphiphilic drugs were notably higher compared to hydrophilic drug molecules when administered via the nasal route in liposomal formulations. This is attributed to their efficient absorption through both direct and indirect transport pathways to the brain [31]. On the other hand, hydrophilic drug molecules with molecular weights less than 1000 Da can be readily delivered to the brain via liposomal formulations when administered intranasally. For instance, higher levels of acetylcholine esterase reactivity were observed when pyridine-2-aldoxime methochloride was administered via intranasal liposomal formulations in poisoned rats [100]. Furthermore, elevated levels of ferric ammonium citrate were noted in the brain when administered intranasally from liposomal formulations [101].

Rats treated with liposomal formulations of donepezil exhibited higher AUC and C_max_ levels in both plasma and the brain, alongside a prolonged half-life, indicating enhanced systemic and brain bioavailability [102]. Quetiapine fumarate administered intranasally via liposomal formulations exhibited markedly higher brain levels for an extended period compared to the free drug, possibly attributable to increased brain uptake via endocytosis. These studies collectively underscore the efficacy of liposomal formulations in delivering drug molecules from the nose to the brain, encompassing both hydrophilic and hydrophobic compounds. However, it is essential to take into account the physicochemical characteristics such as size and surface charges when selecting components for liposome preparation. These factors can influence nasal residence time and mucoadhesion, which in turn can impact the subsequent process of the brain’s uptake of the encapsulated drug molecules [31].

Liposomes, as nanoparticles, may be used in nose-to-brain drug-delivery systems to enhance the precision, effectiveness, and safety of drugs for neurological illnesses such as schizophrenia [103]. Olanzapine is a pharmacological agent used for the treatment of schizophrenia, a mental disorder characterized by abnormal thoughts and behaviors. The oral administration of olanzapine presents difficulties such as limited absorption into the bloodstream and the occurrence of adverse effects throughout the body. However, the liposomal formulation developed showed hopeful options for nose-to-brain transport of olanzapine in the treatment of schizophrenia [104]. One of the brain-distribution studies by Zhao et al. indicated the effectiveness of liposomal formulations as the best carrier for delivering BBB-impermeable drugs to the brain [105]. The authors developed gelatin nanoparticles containing bFGF encapsulated in liposomes, combining the advantages of both polymeric nanoparticles and liposomes. The data showed improved neurological severity score and spontaneous locomotor activities after 3 days of nasal administration of liposomal formulation containing bFGF providing higher levels of growth factor in the hippocampus and pallium areas of the brain as compared to the intravenous administration of solutions or liposomes of bFGF [105]. These data signify the combined advantages of both polymeric nanoparticles and liposomes.

### 7.2. Polymeric Nanoparticles (PNs) for Nose-to-Brain Delivery

Polymeric nanoparticles have been extensively explored for nose-to-brain drug delivery due to their versatility, biocompatibility, and tunable properties. Indeed, the PNs enable the customization of important characteristics like size, shape, surface chemistry, and charge that regulate the interface with the mucous membrane and the molecular and cellular signals controlling its transport. The trigeminal nerve pathway was found to be the primary route for PN absorption in nose-to-brain transport [42]. The potential of PNs in enhancing brain drug delivery via the nose-to-brain route in different neurodegenerative diseases is briefed in a recent review [73]. The significance of biopolymer NPs has demonstrated a higher capacity to deliver medicine to the CNS and to enhance its pharmacological action of actives by nose-to-brain delivery was also reported [74]. Drug molecules including carboplatin, carmustine, diazepam, lorazepam, lurasidone, dolutegravir, and oxcarbazepine have been assessed by developing PNs for brain delivery by intranasal route [106,107,108]. Biodegradable and biocompatible polymers including chitosan, polycaprolactone, polylactic acid, and PLGA (poly lactic-co-glycolic acid), which are approved by the US FDA, were widely utilized in developing PNs aimed for intranasal delivery [109]. In one attempt, a direct nose-to brain delivery of lamotrigine-loaded PLGA PNs was assessed in rats for effective therapy in neuropathic pain [110]. The biodistribution study results signify that the developed NPs are ideal for intranasal application and could be a feasible approach for brain drug delivery via nasal administration.

The potential of PNs for glioblastoma treatment was demonstrated using temozolomide, an anticancer drug used for treating brain tumors whose efficacy is limited due to poor BBB penetration. Researchers have developed drug-encapsulated PNs composed of materials like PLGA or chitosan which could serve as carriers, protecting the drug from degradation and facilitating its transport to the brain [111]. The higher efficacy of naringin-loaded chitosan NPs in brain delivery was reported recently [112]. Indeed, the developed NPs demonstrated a 267-fold reduction in the oxaliplatin-induced chemobrain in rats as compared to oral doses. In targeted drug delivery, the PNs can be surface-modified or functionalized with ligands that enhance their interaction with receptors or transporters in the nasal mucosa. Surface-coated mucus-penetrating PNs are an emerging trend used to increase therapeutic delivery to the brain after intranasal administration. These formulations are expected to minimize quick nasal mucociliary clearance, poor epithelial penetration, and local enzymatic degradation while improving retention. Such observations are demonstrated in a few studies [113,114,115].

An attempt was made to evaluate nose-to-brain transport using surface-modified transferrin PLGA NPs loaded with clonidine [116]. The outcomes demonstrated a statistically significant rise in drug concentration in the brain and improvements in the mice’s behavior (*p* < 0.05) with the developed NPs. Similarly, transferrin chitosan NPs were assessed for their potential to deliver proteins [117]. This study highlights the advantageous properties of such formulation strategies and suggests that a range of targeted ligands could be attached to their surface while preserving the integrity of the cargo. The intranasal delivery of β-Asarone-modified chitosan NPs containing astragaloside IV for multiple sclerosis was reported by a research group [118]. The promising results observed by the authors showed that NPs administered intranasally resulted in preferential brain delivery and were a prospective treatment for multiple sclerosis that could boost the anti-inflammatory and remyelination benefits of astragaloside IV while reducing demyelination. Additionally, studies were published on temozolomide-loaded PLGA NPs functionalized with anti-EPHA3 [111] and baicalin-loaded ligand-modified NPs [119] for different applications.

Despite broad agreement regarding the advantages and potential of the intranasal administration of PNs to target the CNS, along with relatively strong preclinical proofs of concept, this method of treatment still faces significant challenges, which are reflected in the extremely small number of ongoing clinical trials.

### 7.3. Solid Lipid Nanoparticles (SLNs) for Nose-to-Brain Delivery

SLNs are being increasingly studied for their potential to deliver drugs through various routes [120] including the nose-to-brain route. This is because SLNs are known for their stability, biocompatibility, and ability to effectively encapsulate a wide range of pharmaceuticals. The feasibility of SLNs to deliver actives to the brain via the nose-to-brain route was demonstrated using sumatriptan. The prepared SLNs exhibited spherical morphology and sustained drug release up to 12 h. Ex vivo studies indicated quick permeation across nasal mucosa, while histopathology studies confirmed the integrity of the nasal mucosa after treatment [121]. SLNs provide a means to enhance the transportation of drugs to the brain, therefore addressing the issue of drug delivering for the treatment of Alzheimer’s disease. In one study, asiatic acid-loaded SLN was developed with the objective of improving the bioavailability through the intranasal route. The study discovered that learning and memory impairments brought on by amyloid-β_1-42_ were alleviated by the intranasal therapy of drug-loaded SLNs. Moreover, asiatic acid lessened microglial activation and astrocyte reactivity and lowered oxidative stress and inflammatory-marker levels in the brain [122]. A nasal drug-delivery method has been developed by researchers using SLNs that contain agomelatine, an antidepressant drug. The optimized nanoparticles had favorable characteristics and significantly improved plasma concentration, AUC, and absolute bioavailability compared to oral administration. They also demonstrated successful brain targeting, indicating the potential of this approach for enhancing the therapeutic efficacy of agomelatine [123]. Similarly, the SLNs were investigated as a drug-delivery system to enhance the brain-targeting efficiency of rosmarinic acid via intranasal administration. In a rat model of Huntington’s disease, the SLN treatment significantly improved behavioral abnormalities and attenuated oxidative stress. Indeed, the nasal delivery of SLN showed significant therapeutic effects compared to intravenous administration, with a brain drug concentration of 5.69 µg and favorable pharmacokinetic parameters [124]. Another drug, haloperidol, was delivered to the brain through the nasal route. Results of in vitro release studies demonstrated sustained release, while pharmacokinetic studies in rats revealed significantly higher brain concentrations of haloperidol with SLNs compared to other administration routes [125].

The potential of SLNs to produce high local concentrations in the brain during the intranasal administration of the levofloxacin/doxycycline combination was examined. Pharmacokinetic investigations showed that compared to intranasal free solutions, the SLN-based combination considerably increased brain concentrations and AUC in rats. The optimized formulation demonstrated a high proportion of direct transport and drug-targeting efficiency, suggesting that this technique has the potential for brain medication delivery [126]. In another attempt, the hydrogel-based SLN formulation was developed containing n-propyl gallate to improve brain delivery via intranasal administration. The study suggests that hydrogel-based SLNs can enhance nasal absorption by improving mucosal residence time and stability, offering a promising approach for intranasal delivery [127]. Piribedil-loaded SLN in situ gel was developed and evaluated via the nose-to-brain pathway. Pharmacokinetic studies demonstrated that developed gel significantly increased the drug concentration in the brain by approximately 4-fold and reduced plasma concentration by 2.3-fold compared to intranasal suspension. Moreover, the gel system exhibited efficient direct nose-to-brain uptake, with a direct-transport percentage value of 27%, highlighting its potential for enhanced drug delivery to the brain [128]. The SLN-based delivery system for the efficient nose-to-brain transport of BACE1 siRNA for the potential treatment of Alzheimer’s disease was reported. The study results showed enhanced cellular transport of siRNA released from SLNs, across a Caco-2 monolayer, suggesting the potential efficacy of this delivery system [129].

### 7.4. Nanostructured Lipid Carriers (NLCs) for Nose-to-Brain Delivery

NLCs are a sophisticated kind of lipid-based nanoparticle (Figure 2) that have garnered interest for their enhanced drug-loading capacity and stability in the context of nose-to-brain drug administration [79]. NLCs are made to meet all industrial requirements, including quantification, scalability, inexpensiveness, and basic technology. The use of biocompatible and biodegradable lipids and surfactants in NLCs has also made them suitable from a regulatory standpoint. Owing to their lipophilic properties, NLCs exhibit a superior ability to partition into the lipid bilayer of nasal epithelial cell membranes compared to free drugs [130]. Additionally, nanosized particles with ample lipophilicity can easily traverse intercellular spaces between olfactory cells [4]. The inclusion of surfactants in NLCs serves to open tight junctions between epithelial cells, thereby augmenting drug permeability [21]. Research has demonstrated that a polyethylene glycol 25 stearate coating layer inhibits P-glycoprotein efflux at cerebrovascular endothelial cell membranes, leading to increased brain drug concentration [131]. Incorporating drugs into NLCs enhances drug retention in the nasal cavity, with further improvements possible through dispersion in hydrogel systems or coating with mucoadhesive polymers such as chitosan. NLCs also prove more adept at reducing the enzymatic degradation of drugs within nasal mucus compared to traditional solution-based dosage forms. Importantly, the safety profile of NLCs strongly supports their feasibility for nose-to-brain delivery.

Various categories of drugs including agomelatine, aripiprazole, artesunate, asenapine, astaxanthin, diazepam, efavirenz, nicergoline, olanzapine rivastigmine, sumatriptan, etc., have been developed into NLCs to be delivered into the brain through nasal application for targeting CNS disorders. Quetiapine, an antipsychotic medicine, has difficulties in effectively reaching the brain and has adverse effects on the whole body. In one attempt, scientists have developed quetiapine-loaded NLCs for nose-to-brain delivery for the treatment of bipolar disorder. Results demonstrated the superiority of the developed quetiapine-loaded NLC formulation as a stable drug-delivery system. Overall, it was concluded that the NLCs provide a promising approach to enhance the transportation of quetiapine to the brain, hence addressing the treatment of bipolar disorder [132]. The potential of NLCs in delivering diazepam-loaded NLCs by intranasal delivery and the possible toxicity were reported. A QbD approach was used in developing carriers, which essentially provided an ideal NLC formulation for effective brain delivery. This study concludes that the negatively charged NLCs developed were most suitable for nose-to-brain delivery [133]. In another study, NLCs containing an antiretroviral agent (efavirenz) were developed and evaluated for brain targeting via nose-to-brain delivery. Based on their findings, it was suggested that selecting the appropriate lipid carrier and strategically utilizing excipients can be beneficial for efficiently targeting efavirenz to the brain [131]. The potential of in situ gel containing NLC was also reported [134].

The intranasal delivery of asenapine using glycol chitosan-coated NLCs showed promising results, with increased systemic and brain bioavailability compared to asenapine solution. The developed NLCs demonstrated good biocompatibility and could be a potential carrier for intranasal drug delivery, offering improved pharmacokinetics and safety [135]. The potential of NLCs in delivering astaxanthin, a potent antioxidant with anti-inflammatory and neuroprotective effects, to the brain was assessed in rats. It was noticed that the intranasal administration of the developed NLCs significantly reduced oxidative stress, neuroinflammation, and apoptosis while improving cholinergic neurotransmission [136]. Similar studies were also reported by other researchers wherein the NLCs have demonstrated their potential in delivering different actives to the brain via nose-to-brain delivery [137,138,139].

### 7.5. Dendrimers for Nose-to-Brain Delivery

Dendrimers are complex polymers with distinct architectures, wherein pharmaceutical actives can be either physically enclosed within the internal cavities of dendritic molecules or chemically conjugated to the terminal functional groups [140]. Dendrimers have distinct surface functions that enable surface alterations to provide targeted drug delivery, hence enhancing brain targeting [141,142]. They have potential in facilitating drug transportation from the nasal cavity to the brain as a result of their distinctive characteristics [143].

Studies were conducted on rats to examine the effects of different concentrations and generations of polyamidoamine dendrimers on the nasal absorption of insulin, calcitonin, and fluorescein isothiocyanate-labeled dextran [144]. Researchers investigated the therapeutic impact of the intranasal delivery of siRNA targeting high-mobility group box-1 in the postischemic rat brain [145]. The nasal administration of this biodegradable polyamidoamine dendrimer significantly reduced cerebral infarct volume in rats following cerebral ischemia by up to approximately 43%, leading to the recovery from neurological and behavioral deficits. This highlights the considerable potential of nasal gene delivery to the brain using dendrimers. In a different study, scientists examined the effectiveness of a dendrimer-based formulation of haloperidol for targeting the brain through intranasal administration [146]. The developed formulation exhibited significantly greater distribution of the drug in both the brain and plasma compared to a control (intraperitoneal). Furthermore, the nose-to-brain delivery of prepared formulation achieved comparable behavioral responses to those induced by control with a very low dose (~7 times lower). Indeed, these observations highlight the potential of dendrimers to improve drug delivery to the brain. Xie et al. highlighted another intriguing method of delivering paeonol to the brain through the nose utilizing dendrimers via in situ gel [147]. It was found that the developed in situ gel greatly increased the nanocomposites’ nasal transport effectiveness and provided greater drug levels in the brain, indicating its potential for nose-to-brain delivery. Studies have also been carried out to assess the in vivo effects of nasal exposure to polyamidoamine dendrimers on neurological biomarkers in the mouse brain [148]. The results imply that the dendrimers may enter the brain through the olfactory nerve or the systemic circulation following intranasal delivery. They also suggest that dendrimers may potentially cause neuronal effects by modifying the brain-derived neurotrophic-factor signaling pathway’s gene expression following a single intranasal administration.

### 7.6. Polymeric Micelles for Nose-to-Brain Delivery

Micelles are structures formed via amphiphilic molecules (with both hydrophobic and hydrophilic regions) in a solution [149]. They are produced by the spontaneous organization of amphiphilic molecules into core-shell structures in water-based solutions. Micellar formulations are especially preferred for drug delivery because of various benefits including enhanced penetration across biological barriers, thermodynamic stability, ease of formulation, and Newtonian flow properties [150]. Owing to these benefits, micellar nanosystems can be used for a variety of purposes and are also being researched for noninvasive drug-delivery methods like nose-to-brain delivery. The polymeric micelles have shown promise in facilitating the transportation of drugs from the nose to the brain [73]. The feasibility of mixed polymeric micelles to deliver dexamethasone through the nose-to-brain pathway for treating CNS diseases was assessed by Sipos et al. The prepared formulation exhibited a low Z-average and high surface polarity, leading to a 14-fold increase in water solubility and favorable dissolution profiles under nasal and axonal conditions. In vitro studies showed high permeability on polar brain lipid extract, and diffusion studies confirmed efficient passage through the nasal mucosa due to strong mucoadhesive properties [151]. Similarly, a meloxicam-loaded polymeric micelle formulation for nose-to-brain delivery to treat neuroinflammation was reported. The developed formulation showed a significantly improved drug dissolution rate and nasal permeability, indicating its potential for the effective treatment of neuroinflammation through nose-to-brain delivery [152]. Another study focused on developing mixed polymeric micelles of lurasidone for intranasal delivery. The prepared formulation showed favorable characteristics and demonstrated increased drug-targeting efficiency compared to pure lurasidone, indicating the potential of micelles for effective brain delivery via the intranasal route [153].

The potential of lecithin-based polymeric micelles in brain delivery by the intranasal route was demonstrated with clozapine. The prepared carrier exhibited a small particle size, higher entrapment efficiency, and a notable improvement in drug penetration and biodistribution to the brain. This study shows that intranasal self-assembling mixed polymeric micelles based on lecithin can be a useful tactic for brain-targeting clozapine [154]. Another study focused on developing tumor-selective polymeric micelles containing camptothecin for nose-to-brain tumor therapy. Indeed, these nano-micelles exhibited a selective uptake via GRPR-expressing C6 glioma cells, cytotoxic effects against these cells, and specific accumulation in C6 tumor tissue in rats, demonstrating the potential of this approach for brain-tumor therapy [155]. The intranasal administration of olanzapine was evaluated in order to target the brain and minimize systemic adverse effects. Research conducted on a rat model revealed that the nasal injection of the micelles resulted in improved targeting of the CNS and greater anti-schizophrenic effects [156]. Clonazepam-loaded polymeric micelles have been developed for direct brain delivery to treat status epilepticus. The optimized formulation demonstrated good entrapment efficiency, zeta potential, prolonged release, and a low polydispersity index. In vivo investigations demonstrated that the produced micelles had a much higher drug-targeting efficiency, drug-targeting index, and nose-to-brain direct-transport % than intranasal solution, indicating that they could be used as an emergency treatment for status epilepticus [157].

A thermosensitive gel delivery system was developed for rotigotine using polymer micelles to improve brain-tissue concentration. The optimized formulation showed extended mean residence time and increased distribution of the drug in brain regions compared to intravenous administration [158]. The utility of micelles as a potential strategy to deliver siRNA was investigated by developing cell-penetrating peptide-modified nano-micelles. The interesting results here demonstrate that the prepared micelles improved brain delivery, likely due to enhanced transport by different pathways [159].

### 7.7. Nanoemulsions (NEs) for Nose-to-Brain Delivery

NEs have been investigated as a means of administering different drugs to the brain via the nasal route for diverse neurological treatments. NEs are colloidal systems composed of tiny oil droplets scattered in a water-based solution. They have shown great potential as effective carriers for delivering drugs from the nose to the brain [84]. The minute size and durability of NEs facilitate the transportation of drugs over the olfactory pathways, hence augmenting the penetration of actives into the brain [160]. It has been described that the extremely high encapsulation of lipophilic medications in o/w-type NEs provides increased solubility, absorption, and bioavailability while minimizing enzymatic degradation [77]. The formulation techniques for NEs need a meticulous selection of components, including biocompatible oils such as medium-chain triglycerides, surfactants such as polysorbates or sorbitan esters, and cosurfactants like ethanol or propylene glycol. The ratios of these components are fine-tuned to attain stability and droplets at the nanoscale. High-energy techniques, such as ultrasonication or high-pressure homogenization, are used to enhance the precision of droplet size [161].

Hydrophobic medicines such as curcumin or tacrine are added to the oil phase to improve their solubility and stability. The selection of components must prioritize safety and biocompatibility. It has been proven that the intranasal route of administration of NEs containing rivastigmine is more efficient by achieving higher brain concentrations, which could provide significant benefit in terms of improving its therapeutic prospects [162]. Similarly, the potential of kaempferol-loaded NEs was assessed for nose-to-brain delivery. The data presented show that the developed mucoadhesive nanoemulsion is superior in brain targeting while decreasing glioma cell survival by promoting apoptosis, which is an objective of cancer treatment [163]. The ability of NEs to transport lurasidone for the prospective therapy of schizophrenia was also revealed [164]. Other examples of NEs used for delivering drugs from the nose to the brain include curcumin NE, which has anti-inflammatory characteristics [165,166], tacrine NE, used for treating Alzheimer’s disease [167], risperidone NEs, which serves as a delivery mechanism for antipsychotic drugs [168], resveratrol NEs used for neurodegenerative disorders [169], and bromocriptine NE, which enhances brain targeting [170]. These formulations are designed to address the difficulties related to the limited capacity of medications to dissolve and be absorbed by the body, providing a promising approach for the effective transport of pharmaceuticals from the nose-to-brain route. Current research in this area is actively investigating novel formulations and medications to achieve improved treatment results [161].

Levodopa is the primary drug used to treat Parkinson’s disease; however, its capacity to reach the brain and metabolic rate restrict its utility. A technique was devised to build a nasal delivery system with good thermodynamic and oxidative stability by enclosing levodopa in NEs [171]. Using response surface methodology, the researchers investigated the interactions and important factors associated with lecithin and Cremophor EL levels. They concluded that RSM is an effective approach for optimizing levodopa NE formulations, with lecithin and Cremophor EL stabilizing the levodopa-loaded NE.

### 7.8. Nanosuspensions (NSs) for Nose-to-Brain Delivery

NSs, which are colloidal dispersions of drug particles in their pure form, are stabilized by surfactants or polymers [172]. The NS consists of stabilized drug particles that are disseminated in a liquid carrier. This improves the solubility and stability of drugs, preventing the medication from breaking down in the nasal cavity [173]. The NS enhances the targeting of the brain by improving the solubility and dissolution rate of drugs, hence allowing its absorption via the nasal mucosa [174]. The diminutive size of particles and the stabilization facilitated by surfactants or polymers assist in the transportation of drugs, hence augmenting the delivery of drugs to the brain. The use of NSs for nasal administration leads to an elevated concentration of clozapine in the brain, which has the potential to improve its therapeutic benefits in the management of symptoms related to schizophrenia [175].

NSs were prepared using a combination of antiretroviral agents for Neuro-AIDS treatment and evaluated for the potential of intranasal delivery [87]. A recent study suggests that the nose-to-brain administration of efavirenz NS is the best approach for neuro-AIDS therapy [176]. Another experiment produced an NS-based in situ gel that proved to be a practical and efficient nasal preparation for breviscapine delivery [88]. Studies have also shown that ionic-sensitive in situ gel containing resveratrol NS improves nasal mucosal permeability and nasal-cavity residence time [177].

### 7.9. Carbon Nanotubes (CNTs) for Nose-to-Brain Delivery

CNTs have potential in medication delivery owing to their distinctive structure and characteristics. However, their precise use in nose-to-brain delivery systems remains a subject of current investigation. These fibers can avoid the BBB by diffusing through the glial cytoplasmic membrane or passing via the nasal cavity and nerve terminals [178]. CNTs have a large surface area and distinctive structure, which may enable them to traverse the BBB or aid in transportation via olfactory pathways [90]. Functionalized CNTs loaded with neuroprotective peptides have the potential to facilitate the precise distribution to affected areas of the brain after a stroke [179].

Neuroprotective peptides have the ability to diminish brain damage and facilitate healing after a stroke [180,181]. Nevertheless, transporting them to the brain is difficult because of the BBB. Due to their unique features, CNTs might be used as carriers for these peptides in order to enhance their transportation to the brain after a stroke [89,181]. Studies on rats and fish revealed that olfactory neurons in the nose can take up nanosized carbon particles and translocate them to the brain [182]. Scientists have modified CNTs to transport neuroprotective peptides and developed a drug-delivery system that is administered via the nose. One of the investigations has demonstrated the potential of CNTs to enter the brain when administered intranasally, specifically reaching the limbic area, which is important for the onset and course of many neurodegenerative illnesses [183]. Additionally, they showed that electroconductive multi-walled carbon nanotubes could alleviate neurodegeneration-related gliosis and potentially modulate a crucial neurotrophic factor to have neuroprotective effects.

Through the use of CNTs for nasal administration, the improved targeting of neuroprotective peptides to the brain has the potential to decrease brain damage and assist in the preservation of neurons after a stroke, hence facilitating recovery and minimizing neurological impairments [184]. This hypothetical scenario demonstrates the possible use of CNTs as vehicles for neuroprotective peptides in the treatment of stroke. On the other hand, the possible toxicity of multiwalled CNTs when delivered via the olfactory route to the brain was documented [185]. Nevertheless, it is crucial to acknowledge that this application falls within the domain of theoretical investigation, and further study is required to authenticate the safety, effectiveness, and precise processes of CNTs in transporting medicinal substances to the brain via the nasal pathway.

### 7.10. Mesoporous Silica Nanoparticles (MSNs) for Nose-to-Brain Delivery

MSNs have a honeycomb-like porous structure containing numerous pore channels (known as mesopores) that have the ability to absorb molecules. MSNs have attracted attention in the field of drug administration because of their adjustable pore diameters, large surface area, and compatibility with living organisms [186]. MSNs have been used as a targeted delivery system for delivering therapeutic agents to various organs [187]. The potential of ponatinib-loaded molecular-gated mesoporous nanoparticles to enhance brain delivery in glioblastoma by intranasal delivery has been reported [188]. It was found that the developed formulation was nontoxic for BBB cells, while a greater amount of the drug was successfully delivered into the brain. Multiple sclerosis is characterized by inflammation in the CNS. Delivering anti-inflammatory drugs directly to specific parts of the brain that are afflicted is a difficult task. MSNs have the potential to function as carriers for drugs to alleviate inflammation in people with multiple sclerosis [189]. Their distinct characteristics provide regulated medication release, perhaps facilitating the medicine’s access to brain areas impacted by inflammation in multiple sclerosis [190]. The potential of MSNs to improve the brain delivery of hydrophobic phytochemicals through the nasal route has been demonstrated [91]. The use of MSNs for delivering corticosteroids via the nose might improve the targeting of these drugs to the brain. This could reduce inflammation in the CNS, providing a targeted anti-inflammatory impact while limiting adverse effects that affect the whole body. Nevertheless, much investigation is required to confirm the safety, effectiveness, and exact processes of MSNs in medication delivery systems from the nose-to-brain route for neurological disorders [91,92].

### 7.11. Nanogels for Nose-To-Brain Delivery

Nanogels are a flexible and promising drug-delivery system for delivering medications via the nose-to-brain route [191,192]. They possess a distinct set of characteristics including a high water content, biocompatibility, and the capacity to enclose many types of pharmaceuticals. The nanoscale hydrogel structures, with sizes generally ranging from 10 to 200 nm, are produced by chemically connecting polymer chains together. This process forms a three-dimensional network that effectively captures medicinal compounds. Regarding the distribution of substances from the nose to the brain, nanogels have many benefits such as prolonged release, the safeguarding of enclosed medications from deterioration, and the possibility of delivering them specifically to the CNS [191,192].

The process of developing nanogels for medication transport from the nose to the brain requires a meticulous evaluation of several crucial elements. Choosing the right polymers is of utmost importance, with a preference for biocompatible and mucoadhesive polymers that can improve the duration of contact with the nasal mucosa. Moreover, the inclusion of hydrophilic and hydrophobic monomers enables the entrapment of a wide array of medications, addressing various therapeutic requirements. The dimensions, electrical charge, and surface characteristics of the nanogel may be customized to enhance its interactions with the nasal mucosa, hence facilitating the efficient administration of drugs [33].

The nanogel is often made up of biocompatible and mucoadhesive polymers such as chitosan and polyethylene glycol (PEG). Due to its cationic nature, chitosan has the ability to interact with the negatively charged nasal mucosa, resulting in an extended period in which it remains in the nasal cavity. PEG enhances the water-attracting properties and durability of the nanogel. The interconnected arrangement of the nanogel safeguards the delicate nerve-growth factor, shielding it from deterioration while in transport. The nanogel’s compact dimensions promote effective absorption via the nasal cavity, while its mucoadhesive characteristics improve its contact with the mucosal surface [93]. When administered nasally, this nanogel containing nerve-growth factor may effectively cross the BBB and enter the brain parenchyma. The nanogel’s sustained release capabilities guarantee a longer therapeutic impact, making it a desirable choice for illnesses that benefit from continued drug administration, such as neurological disorders [94]. Various categories of drugs have been formulated as nanogels and evaluated for their efficacy by intranasal delivery in different conditions including Alzheimer’s, schizophrenia, migraine, hypertension, depression, and cancer [33].

## 8. Nanoparticle Toxicity to Nasal Mucosa

The use of nanoparticles in drug-delivery systems for nose-to-brain applications shows enormous potential but also raises concerns about their possible toxicity to the sensitive nasal mucosa. Their diminutive dimensions and varied surface characteristics facilitate effective engagement with biological tissues, hence increasing the potential for deleterious consequences. The toxicological profile of nanoparticles is greatly influenced by factors such as their size, surface charge, and composition [193]. Submicron particles have the ability to enter cellular structures, which may have an effect on the functioning of cells. Additionally, alterations to the surface properties and chemical makeup of these particles can initiate inflammatory reactions or cause cellular stress. Non-biodegradable nanoparticles have the ability to collect in the mucosa, which might result in extended exposure and possible damage [194]. Mitigation measures include the development of biocompatible materials, the optimization of surface features to limit unfavorable interactions, and the investigation of biodegradable nanoparticles to decrease the potential for accumulation. Thorough preclinical evaluations, which include conducting tests in a controlled environment and using animal subjects, we can carefully examine the potential harmful effects of nanoparticles. The goal is to guarantee that nasal drug delivery is both safe and effective, while also maintaining the integrity of the nasal mucosa. To ensure the safe and efficient transport of drugs from the nose to the brain using nanoparticles, it is crucial to have a thorough knowledge of their possible hazardous effects. Additionally, it is important to use creative design strategies to minimize any dangers that may be connected with their use [195].

## 9. Challenges to Overcome in Nanoparticle-Derived Toxicity to Nasal Mucosa

In an effort to address concerns regarding the potential harmful effects of nanoparticles on the nasal mucosa in drug-delivery systems, various strategic methods are being employed to minimize negative impacts and enhance safety. An essential approach involves the careful and precise design and engineering of nanoparticle features, including size, surface qualities, and composition. Biocompatible materials and surface modifications, such as coatings or functional groups, are specifically designed to minimize interactions that might potentially cause inflammation or cellular stress [90]. Nanoparticle safety and biocompatibility are comprehensively assessed through in vitro investigations and animal models. The purpose of these evaluations is to understand cellular reactions, inflammatory responses, and potential effects on the integrity of mucosal tissues. The knowledge acquired from this research is used to make appropriate modifications in the design and composition of nanoparticles in order to guarantee safety while maintaining effectiveness [196]. To effectively address the issue of nanoparticle toxicity to the nasal mucosa, a comprehensive strategy necessitates the meticulous manipulation of nanoparticle characteristics, the incorporation of biodegradable components, and thorough preclinical assessments. By applying these principles/alterations, researchers can develop drug-delivery systems designed to target the brain through the nasal route specifically, while minimizing potential hazards to the delicate nasal mucosa [197].

## 10. Future Strategies for Nose-to-Brain Delivery

The discipline of nose-to-brain drug administration is expected to see significant progress in the coming years, thanks to novel approaches that seek to overcome existing constraints and improve the effectiveness of treatments. Expected advancements include the enhancement of carriers based on nanotechnology, with the aim of developing nanoparticles that are more accurate and versatile, capable of delivering drugs in a customized manner and monitoring diagnostics. Biomimetic nanoparticles, which draw inspiration from biological architecture, provide the potential for enhanced biocompatibility and diminished immune reactions, hence assuring the safety of delivery systems. Progress in precision medicine has the potential to introduce a new period of tailored treatments, adapting medication compositions to suit each patient’s needs for enhanced effectiveness and reduced adverse reactions [197]. Integrating therapeutics and diagnostics in theranostic platforms has the potential to revolutionize treatment monitoring and delivery, allowing for the immediate evaluation of medication response. Intelligent, bioresponsive delivery systems that are engineered to release drugs in response to particular biological signals have the potential to provide precise and timely drug release. Novel formulation strategies are being studied to improve stability, boost mucoadhesion, and increase drug-loading capabilities. These approaches aim to provide greater retention and prolonged release of drugs in the nasal cavity [46]. Furthermore, the advancement of gene and RNA treatments for direct delivery to the brain by the nose shows potential for precise interventions at the genetic level. Simultaneously, efforts are focused on discovering novel biological agents that may promote the transportation of drugs, therefore increasing their capacity to pass through the nasal mucosa and improving their absorption and availability in the body [53]. In summary, these upcoming solutions aim to revolutionize the delivery of drugs from the nose-to-brain route, offering more precise, efficient, and personalized treatments. With these advancements, the field is poised to overcome existing obstacles, expand the spectrum of treatable neurological disorders, and significantly improve patient outcomes [198].

## 11. Conclusions

This review summaries the significant advancements and innovations in the field of nose-to-brain drug delivery while depicting the recent developments highlighting the anatomical features of the nasal cavity, emphasizing its high permeability and rich blood supply that facilitate rapid drug absorption and onset of action, rendering it a promising domain for neurological therapeutics. Fundamentally, various factors are required to be considered while developing nose-to-brain drug-delivery systems. The major challenges in this drug-delivery system include nasal architecture, mucociliary clearance, drug properties, formulation stability, and targeting efficiency, etc. Apart from solutions, various other dosage forms including nanocarriers and nanogels, etc., can be among the alternative approaches for safer and more efficient nose-to-brain drug delivery. Numerous investigations have shown that the right nanocarrier can dramatically boost the dosage that reaches brain tissue. Targeting specificity and thereby improving the drug-delivery efficiency of such advanced delivery systems will revolutionize the future of neurological therapies. Recent studies have attempted to translate the laboratory results into clinical applications, although with varying degrees of success. However, the novelty of this approach and administration route means that little has been done in terms of optimizing and improving dosing efficiency. Key challenges include the limited volumes that can be administered, variations in the nasal anatomy of animals studied and humans, which can affect the clinical outcomes, and the necessity for developing formulations and suitable devices for this route of administration, and to overcome nanoparticle-dependent toxicity to the nasal mucosa and to the brain tissue.

## Figures and Tables

**Table 1:** Anatomical and physiological features of various nasal-cavity regions.

Aspect	Description	Reference
Nasal Cavity	Hollow space behind the nose	[1]
Nasal Septum	Wall of cartilage dividing the nasal cavity	[18]
Nasal Turbinates	Curved bony structures that help filter and humidify air	[19]
Mucous Membrane	Lines the nasal cavity, secretes mucus	[7]
Cilia	Tiny hair-like structures that help move mucus	[5]
Blood Supply	Rich vascular network supplying the nasal passages	[6]
Functions	Filtration, humidification, and warming of inhaled air	[5,6]
Olfactory	Houses receptors for the sense of smell	[13,14]
Sinuses	Air-filled cavities connected to nasal passages	[20]
Airflow	Inhaled air passes through the nostrils into the cavity	[16]
Physiology	Mucus traps particles: cilia move them to the throat	[17]
	Air is warmed and humidified before reaching the lungs	[16,17]
	Receptors that sense smell perceive and analyze odors.	[17]

**Table 2:** Exploring the anatomical and physiological regions of the nasal cavity: advantages, challenges, mechanisms, and applications.

Aspect	Description	Reference
Olfactory Region	Area in the nasal cavity housing olfactory receptors	[27]
Olfactory Epithelium	Specialized tissue containing olfactory sensory neurons	[27]
Nasal Mucosa	Lined with blood vessels and nerve endings	[7]
Nasal Pathways	Direct connection between the nasal cavity and brain	[8]
Mechanisms of Delivery	Nasal epithelium absorption	[9]
	Olfactory nerve pathway	[9]
	Lymphatic transport	[28]
Delivery Vehicles	Intranasal sprays	[29]
	Nanoparticles	[30]
	Liposomes	[31]
	Micelles	[32]
	Hydrogels	[33]
Advantages	Rapid onset of action	[34,35]
	Bypasses blood–brain barrier	[35,36]
	Reduced systemic side effects	[37]
Applications	Drug delivery for neurological disorders	[38]
	Treatment of brain tumors	[39]
	Targeted therapy for neurodegenerative diseases	[34,35]
Challenges	Nasal mucosa variability	[35]
	Clearance mechanisms	
	Dosage control and optimization	
	Safety concerns, potential irritation	

**Table 3:** Various nanoparticles with their characteristics for nose-to-brain drug-delivery systems.

Nanoparticle Type	Description	Mechanism of Action	Advantages	Challenges	Applications	Reference
Liposomes	Spherical vesicles composed of lipid bilayers, enclosing an aqueous core. They can encapsulate hydrophilic and lipophilic drugs simultaneously.	Facilitates drug solubility and stability. Enhanced drug delivery due to biocompatibility with cell membranes. Controlled release profiles.	Versatile drug encapsulation. Biocompatible and non-toxic. Targeted drug delivery.	Short shelf life due to stability issues. Large-scale production. Potential immune response.	Treatment of brain tumors. Neurodegenerative disorders. Brain infections. For example, Olanzapine, Donepezil, Quetiapine, and Resperidone.	[31,69,70,71]
Polymeric NPs	Nanoparticles made from biodegradable polymers like PLGA, chitosan, or PEG. They can encapsulate drugs and protect them from degradation.	Controlled drug release. Improved drug stability. Targeted delivery to brain regions.	Controlled drug release. Biocompatible and biodegradable. Enhanced drug stability.	Reproducibility and standardization. Potential toxicity of polymers. Variability in drug-loading efficiency.	Treatment of neurodegenerative diseases. Brain tumor therapy. CNS infections. For example, Baclofen, Lamotrigine, Tapentadol, and Timozolemide.	[72,73,74,75]
Solid lipid nanoparticles	Nanoparticles composed of lipids offer a solid matrix for drug encapsulation, enhancing drug stability and sustained release.	Enhanced drug stability. Controlled drug release. Increased bioavailability.	Improved drug stability. Sustained drug release. Biocompatible.	Maintaining a solid state during storage. Limited drug-loading capacity. Potential lipid oxidation.	Drug delivery for brain disorders. Brain tumor therapy. Neurodegenerative diseases. For example, Agomelatine, Asiatic acid, Haloperidol, Ondansetron, Piribedil, and Risperidone.	[76,77,78]
Nanostructured lipid carriers	Improved versions of SLN with enhanced drug-loading capacities, preventing drug expulsion and offering controlled release.	Higher drug-loading capacity. Enhanced drug stability. Controlled and sustained drug release.	Increased drug-loading capacity. Improved stability. Controlled drug release.	Maintaining stability during storage. Potential lipid oxidation. Complexity in production methods.	Treatment of brain disorders. Brain tumor therapy. Neurodegenerative diseases. For example, Agomelatine, Asenapine, Astaxanthin, Diazepam, Efavirenz, Olanzapine Rivastigmine, and Sumatriptan.	[4,79,80,81]
Dendrimers	Highly branched, symmetrical nanoparticles with well-defined structures. They offer multifunctionality and precise control over size and surface properties.	Precise control over structure and properties. High drug-encapsulation efficiency. Targeted drug delivery.	Tailored surface properties. High drug-loading capacity. Precise control over structure.	Potential toxicity concerns. Complex synthesis methods. Large-scale production.	Treatment of brain tumors. Neurodegenerative disorders. CNS infections. For example, SiRNA, Haloperidol, and Paeonol.	[73,82]
Polymeric micelles	Self-assembling colloidal nanoparticles formed by amphiphilic molecules in aqueous solutions, encapsulating hydrophobic drugs within their core.	Solubilization of hydrophobic drugs. Increased drug stability. Controlled drug release.	Solubilization of hydrophobic drugs. Biocompatibility. Controlled drug release.	Maintaining stability during storage. Potential drug leakage. Limited drug-loading capacity.	Treatment of brain tumors. Neurodegenerative disorders. Brain infections. For example, Camptothecin, Clonazepam, Clozapine, Dexamethasone, Efavirenz, Lurasidone, Meloxicam, and Olanzapine.	[73,83]
Nanoemulsions	Thermodynamically stable emulsions with droplet sizes in the nanometer range provide enhanced solubility and stability for drug delivery.	Enhanced drug solubilization. Improved drug stability. Controlled drug release.	Improved drug solubility. Enhanced stability. Controlled drug release.	Long-term stability. Complexity in production. Limited drug loading capacity.	Treatment of brain tumors. Neurodegenerative disorders. CNS infections. For example, Clozepine, Lurasidone, Rotigotine, Ropinirole, Naringenin, Ibuprofen, etc.	[84,85,86]
Nanosuspensions	Dispersions of nanosized drug particles stabilized in a liquid carrier, providing improved drug solubility and bioavailability.	Enhanced drug solubility. Improved bioavailability. Controlled drug release.	Enhanced drug solubility. Improved bioavailability. Controlled drug release.	Stability during storage. Limited drug loading capacity. Potential particle aggregation.	Treatment of brain tumors. Neurodegenerative disorders. Brain infections. For example, Rotigotine, Ritonavir, Resveratrol, Efavirenz, and Breviscapine Lopinavir.	[87,88]
Carbon nanotubes	Tubular structures composed of carbon atoms, offering high surface area and unique properties for drug-delivery applications.	Distinctive structure and large surface area. Good transporter. Unique characteristics.	High surface area for drug loading. Potential for targeted delivery. Improve stability.	Toxicity and biocompatibility. Potential immune response. Complex functionalization methods.	Treatment of brain tumors. Neurodegenerative disorders. CNS infections.	[89,90]
Mesoporous silica nanoparticles	Porous nanoparticles with tunable pore sizes and large surface areas for drug loading and release.	Adjustable pore diameters. Good carrier. Surface modification for targeting.	High drug loading capacity. Controlled drug release. Tailored surface properties.	Reproducibility and standardization. Potential toxicity concerns. Controlled particle size and pore distribution.	Treatment of brain tumors. Neurodegenerative disorders. Brain infections. For example, Phytochemicals and Ponatinib	[91,92]
Nanogels	Colloids-based hydrogel with three-dimensional polymeric networks capable of encapsulating drugs and allowing for sustained release.	High water content. Efficient administration. Colloidal particle size	Controlled drug release. High drug-loading capacity. Biocompatible.	Structural stability. Controlling drug release kinetics. Potential toxicity of polymers.	Treatment of brain tumors. Neurodegenerative disorders. CNS infections. For example, Insulin.	[33,93,94]

## Data Availability

The data presented in this study are contained within this article.
